# An Innovative Workshop Embedding Pathology Service Users into the Undergraduate Biomedical Science Curriculum

**DOI:** 10.3389/bjbs.2023.11584

**Published:** 2023-08-08

**Authors:** Amreen Bashir, Kayleigh Wilkins, Ross Pallett

**Affiliations:** School of Biosciences, College of Health and Life Sciences, Aston University, Birmingham, United Kingdom

**Keywords:** patient care, biomedical science, higher education, reflective writing, service users

## Abstract

The integration of pathology service users into the biomedical science curriculum has been driven by the refinement of the Health and Care Professions Council (HCPC) Standards of Proficiency. This study aimed to design and implement a novel and innovative service user event with a reflective assessment to enhance students’ knowledge and understanding of the impact of pathology laboratory results on the patient pathway. The 4-h workshop consisted of a series of service users. Patients discussed how pathology services had contributed to their diagnosis and treatment, while service providers—a Microbiology Consultant, a director of primary care, and the patient referral optimisation officer—discussed their roles and their interactions with pathology services. Post-event, students completed a 750-word reflective assessment, highlighting challenges experienced by service users and providing suggestions for improving the delivery of pathology services. In total, 57.5% of respondents (57/99) completed a post-reflection survey, which included open- and closed-ended questions. Quantitative analysis of the survey data revealed that over 87.7% of respondents had increased knowledge and understanding of the revised HCPC standards. Following the assessment, students reported a significant increase in their confidence with respect to reflective writing (*p* < 0.001), with over 90% of respondents agreeing that the reflective assessment had increased their knowledge and understanding of the limitations that may negatively impact service users and patient care. Moreover, respondents highlighted how advancements in point-of-care testing (POCT) and improvements in communication can improve patient experiences. Thematic analysis revealed that respondents agreed that embedding patients into the curriculum reinforced the importance of there being a patient behind every sample. Respondents reported that reflecting upon service user experiences enabled them to identify improvements to the delivery of pathology services while recognising the essential role that Biomedical Scientists play in the patient pathway. This successful workshop has created a platform encompassing a range of pathology service users in the undergraduate curriculum. We recommend that other accredited biomedical science programmes adopt and embed this innovative workshop and reflective assessment into their programmes to help them meet these standards relating to service users while fostering important transferable skills in their students.

## Introduction

To ensure the delivery of high-quality patient care and pathology services, it is imperative to have a thorough understanding of the needs of patients. The integration of service users into the biomedical science curriculum has been driven by the refinement of the Health and Care Professions Council (HCPC) Standards of Education and Training (SETs), which explicitly state that “service users and carers must be involved in the programme (SET 3.7)” and “the learning outcomes must ensure that learners meet the Standards of Proficiency for the relevant part of the register (SET 4.1)” [1, 2]. The HCPC Biomedical Scientist Standards of Proficiency (SOPs) have underscored the importance of incorporating patients’ perspectives and listening to patients’ voices to enhance the delivery of pathology services and patient care [3]. While the HCPC’s definition of “service user” refers to individuals who utilise or are impacted by the services of HCPC professionals, it has historically been challenging to define pathology “service users,” as pathology laboratories were typically located at the periphery of hospitals with limited interaction with the ultimate service user [1–3].

Since 2014, the HCPC has required all programmes approved by the regulatory body to involve “Service users and carers” in the programme [2]. However, the revised HCPC Standards of Proficiency of September 2023 have emphasised the “central role of the service user” and the requirement for “registrants to understand the importance of valid consent and effective communication in providing good care.” In addition, registrants should be “promoting public health and preventing service users’ ill-health” and understand “the importance of valid consent and effective communication in providing good care” [3]. The timing of these revisions coincides with a shift in public knowledge, where patients now have a better understanding and a greater appreciation of the role of laboratory medicine in the diagnosis and treatment of disease [4].

The COVID-19 pandemic served as a catalyst for raising public awareness and recognition of the critical role played by Biomedical Scientists (BMS) in the United Kingdom in the processing and testing of COVID-19 samples [4]. Before the pandemic, patients were primarily familiar with the role of medical professionals such as doctors and nurses in providing healthcare services, whereas the pandemic drew attention to the vital role of laboratory workers who operate behind the scenes in testing and diagnosing diseases [5, 6].

The evolution of point-of-care testing (POCT) in the last decade has brought about significant changes to the role of Biomedical Scientists as diagnostic testing has become more accessible across healthcare pathways. Many commercial POCT manufacturers recognise the value of close working relationships with BMS and have established collaborative working and development groups [7]. However, the COVID-19 pandemic has dramatically changed the role and responsibilities of BMS, thereby necessitating a corresponding adaptation in the training of future biomedical science students [8]. A BMS processes hundreds of patient samples on a typical workday, which can lead to a lack of appreciation for the fact that each sample represents an individual patient. Thus, it is imperative for biomedical science students to be conscious of the importance of test results for patients. It is important to recognise that medical professionals, such as doctors and nurses, who order laboratory tests are considered service users for pathology laboratories; however, the primary beneficiaries are ultimately the patients themselves.

The involvement of patients in medical education has become a standard practice among educators [9, 10]. The General Medical Council (GMC) has long recognised the value of patient involvement and requires educators to incorporate a variety of patient-centred sessions into the undergraduate curriculum [11]. However, there is still much to be learned about how to systematically integrate patient involvement into other allied healthcare courses. Studies have demonstrated that both patients and practitioners benefit from a patient-centred curriculum [12]. Patients take on the role of educators, teaching students about patient-centred care and the importance of patient autonomy, and helping to make education increasingly engaging and transformative [12]. As BMSs rarely interact directly with patients on a daily basis, the involvement of patients in the curriculum reinforces the importance of the patient being behind every sample.

Medical educators and patients have joined forces in promoting patient-centeredness; however, BMS service users have yet to be fully integrated into the biomedical science curriculum in the same way. Reflecting upon the experience of patients can assist learning and professional development; this reflective writing is considered a core element in medical education that promotes critical thinking, better communication, and empathy skills [13, 14]. Therefore, the aim of this study was to embed patients and BMS service users into the undergraduate biomedical science curriculum through a “service user event” with a reflective assessment to enhance students’ knowledge and understanding of the impact of pathology laboratory results on the NHS service and ultimately the patient.

## Materials and Methods

**FIGURE 1:**
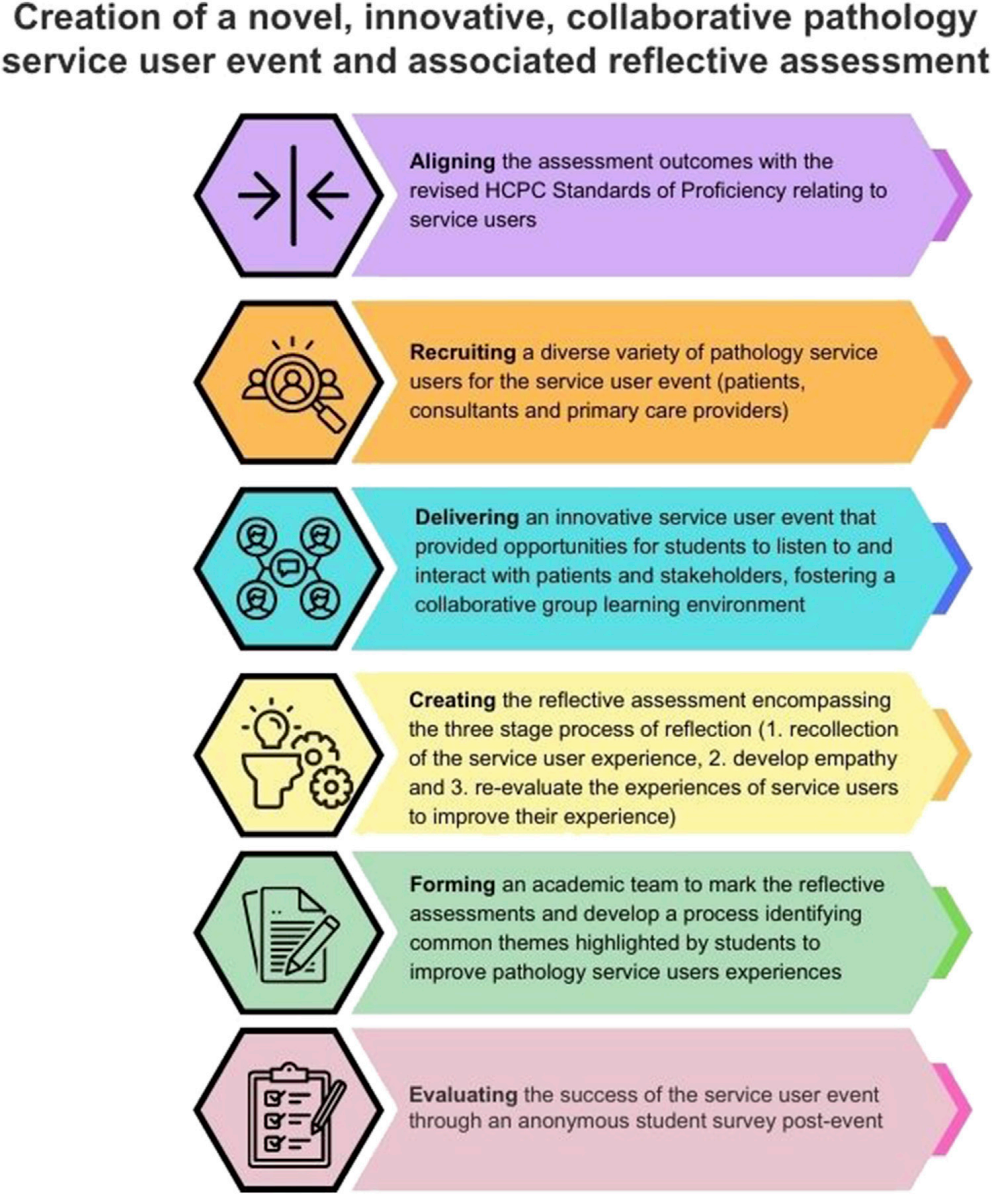
The six-step process involved in creating an event to involve pathology service users in the undergraduate biomedical science curriculum.

The steps involved in the creation of the novel, innovative service user event are detailed in Figure 1 and can be adopted by other higher education institutes that require the incorporation of service users into their curriculum.

### Service User Event

A service user event workshop was created and facilitated by academics from the School of Biosciences, Aston University, United Kingdom, for final-year biomedical science students. The event is part of the 30-credit final-year “Professional Development for Biomedical Scientists” module. The workshop was scheduled for 4 hours, and the following pathology service users were invited as guest speakers: a patient with beta-thalassaemia major, a patient diagnosed with a giant cell tumour, a Microbiology Consultant, a director of a primary care provider, and a patient referral optimisation officer. Both patients and service users provided consent to participate in the workshop. The patients discussed how pathology services have contributed to their diagnosis and treatment, while service providers discussed their roles and their interactions with pathology services. All speakers highlighted issues that have affected the delivery of an optimal service. This was followed by an interactive class discussion, which was directed by the assessment brief.

### Service User Reflection Assessment

Following the event, all students were required to complete a 750-word reflective piece, with this assessment contributing to 33% of the overall module mark (Supplementary Table S2: Assessment Brief). This assessment required students to reflect on the voices of the different service users and how taking action to address problematic areas in healthcare can enhance the delivery of biomedical science and improve patient care. Students were directed to specifically comment on several areas, such as 1) past and current challenges to the delivery of pathology services; 2) advancements in point-of-care testing (POCT) and its increased use in the diagnosis and monitoring of disease; 3) the changing role of Biomedical Scientists and diagnostic laboratories in healthcare; 4) the increased awareness of the profession following COVID-19; and 5) empowering patients to understand and access their results.

To support students in writing a reflective piece, they were provided with a range of resources, which included a workshop that gave them an opportunity to approach reflective writing and links to the marking scheme. Students were also given a generic example of a reflection and were asked to work in mixed groups to mark the reflective piece and provide feedback according to the assessment marking scheme. In addition to this, students were directed to additional reading that covered the importance of reflective writing for practitioners and how to write in-depth reflections. Students were also allowed to attend an additional drop-in session to ask any questions they had regarding the assessment (Supplementary Table S3: Marking Rubric).

### Collecting Student Feedback and Analysing the Results

Final year biomedical science students’ experiences of the service user event were collected following submission of the reflective assessment through an eight-item online questionnaire [15] (Supplementary Data Sheet S1). Ethical approval was granted by the Health and Life Sciences Ethical Committee (Project #1494). Students were invited to participate in the study by email and were provided with a link to the online survey via the virtual learning environment. Online consent was required before accessing the questions. Students completed questions asking whether, after the submission of their reflection, they had an increased understanding of:

(1) The impact of pathology results on service users and effective communication in providing patient care.
(2) An understanding of the changing role of the Biomedical Scientist in the patient pathway.
(3) The value of embedding patients in the biomedical science curriculum to improve the delivery of healthcare.
(4) The value of continuous reflective practice and its role in asking difficult questions and finding meaningful answers.

The questions reflected the revised 2023 HCPC Standards of Proficiency, which address embedding service users within the biomedical science curriculum (Table 1). A mixed methodology approach was adopted, which included open- and closed-ended questions. The results were analysed both quantitatively and qualitatively. To compare the responses of biomedical science students pre- and post-completion of the reflective assessment, a Chi-squared test was used to determine statistical significance (*p* < 0.05). Free-text responses were analysed using thematic analysis [16, 17]. The researchers read the data for familiarity, generated codes to form initial themes, and checked for plausibility. The process was repeated by all three members of the team, and the final themes were collectively agreed upon to produce the thematic analysis.

**TABLE 1:** Survey questions asked following the service user event and reflective assessment and the relevant revised HCPC Standards of Proficiency.

Question: “Following the service user event and reflection assessment I now have increased knowledge and understanding of … ”	Relevant revised HCPC Standards of Proficiency
Public health and prevention of service users’ ill-health	SOP 15.1 understand the role of their profession in health promotion, health education and preventing ill-health
	SOP 15.2: understand how social, economic and environmental factors (wider determinants of health) can influence a person’s health and well-being)
	SOP 15.3: empower and enable individuals (including service users and colleagues) to play a part in managing their own health
The role of equality, diversity, and inclusion, with specific importance placed on ensuring practice is inclusive for all service-users	SOP 5: recognise the impact of culture, equality and diversity on practice and practise in a non-discriminatory and inclusive manner
	SOP 5.1: respond appropriately to the needs of all different groups and individuals in practice, recognising this can be affected by difference of any kind including, but not limited to, protected characteristics, intersectional experiences and cultural differences
	SOP 5.3: recognise the potential impact of their own values, beliefs and personal biases (which may be unconscious) on practice and take personal action to ensure all service users and carers are treated appropriately with respect and dignity
The central role of the service-user, including the importance of valid consent and effective communication in providing good care	SOP 7.1: use effective and appropriate verbal and non-verbal skills to communicate with service users, carers, colleagues and others
	SOP 7.5: modify their own means of communication to address the individual communication needs and preferences of service users and carers, and remove any barriers to communication where possible
	SOP 7.8: understand the need to provide service users or people acting on their behalf with the information necessary in accessible formats to enable them to make informed decisions
The importance of leadership at all levels of practice	SOP 8.6: understand the qualities, behaviours and benefits of leadership
	SOP 8.7: recognise that leadership is a skill all professionals can demonstrate
	SOP 8.8: identify their own leadership qualities, behaviours, and approaches, taking into account the importance of equality, diversity and inclusion
	SOP 8.9: demonstrate leadership behaviours appropriate to their practice
The need to be able to use information, communication and digital technologies appropriate to practice	SOP 6.5: recognise that the concepts of confidentiality and informed consent extend to all mediums, including illustrative clinical records, such as photography, video and audio recordings and digital platforms
	SOP 7.7: use information, communication and digital technologies appropriate to their practice
	SOP 9.3: use digital record-keeping tools where required
	SOP 13.1: be able to change their practice as needed to take account of new developments, technologies and changing contexts

## Results

A total of 99 students were enrolled onto the module, and all attended the service user workshop, 57 of whom completed the post-event online survey. To better understand the demographics of the student cohort, they were asked if they had worked in the NHS in the last 3 years. A total of 20 (35.1%) respondents stated that they had worked in the NHS, with 9% of respondents having completed their Institute of Biomedical Science (IBMS) registration portfolio as a Trainee Biomedical Scientist during their placement year. Other roles included: Medical Laboratory Assistant, Administration and Clerical Staff, Domestic Assistant, Dental Receptionist, Vaccination Support Officer, and Clinical Trial Support Officer.

### Incorporation of Revised HCPC Standards of Proficiency

**FIGURE 2:**
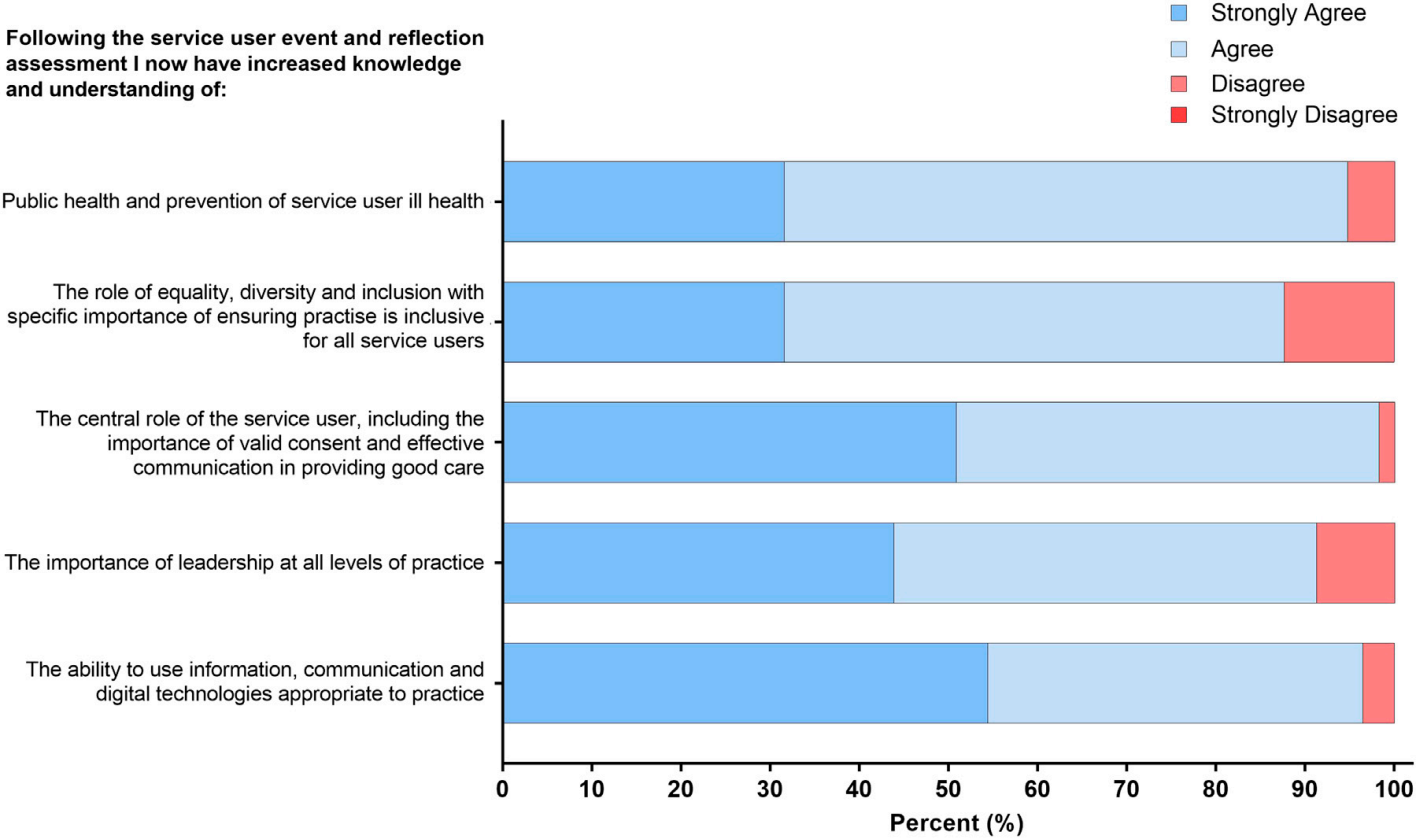
Student responses to statements relating to increased understanding and knowledge of HCPC standards following the service user event. The response to the four-point Likert scale for each statement is shown as a percentage.

Following the service user event, students were asked to reflect on whether their knowledge and understanding of the some of the revised 2023 HCPC Standards of Proficiency for all 15 HCPC registered professions had improved. Of the respondents, an overwhelming percentage either “strongly agreed” or “agreed” that the session increased their knowledge and understanding of; “*public health and prevention of service users’ ill-health*” (94.8%); “*the role of equality, diversity, and inclusion, with specific importance placed on ensuring practice is inclusive for all service-users*” (87.7%); *“the central role of the service-user, including the importance of valid consent and effective communication in providing good care”* (98.3%); *“the importance of leadership at all levels of practice”* (91.3%) and *“the need to be able to use information, communication and digital technologies appropriate to practice”* (96.5%) (Figure 2).

### Reflective Writing Can Emphasise the Central Role of Service Users Within the NHS

**FIGURE 3:**
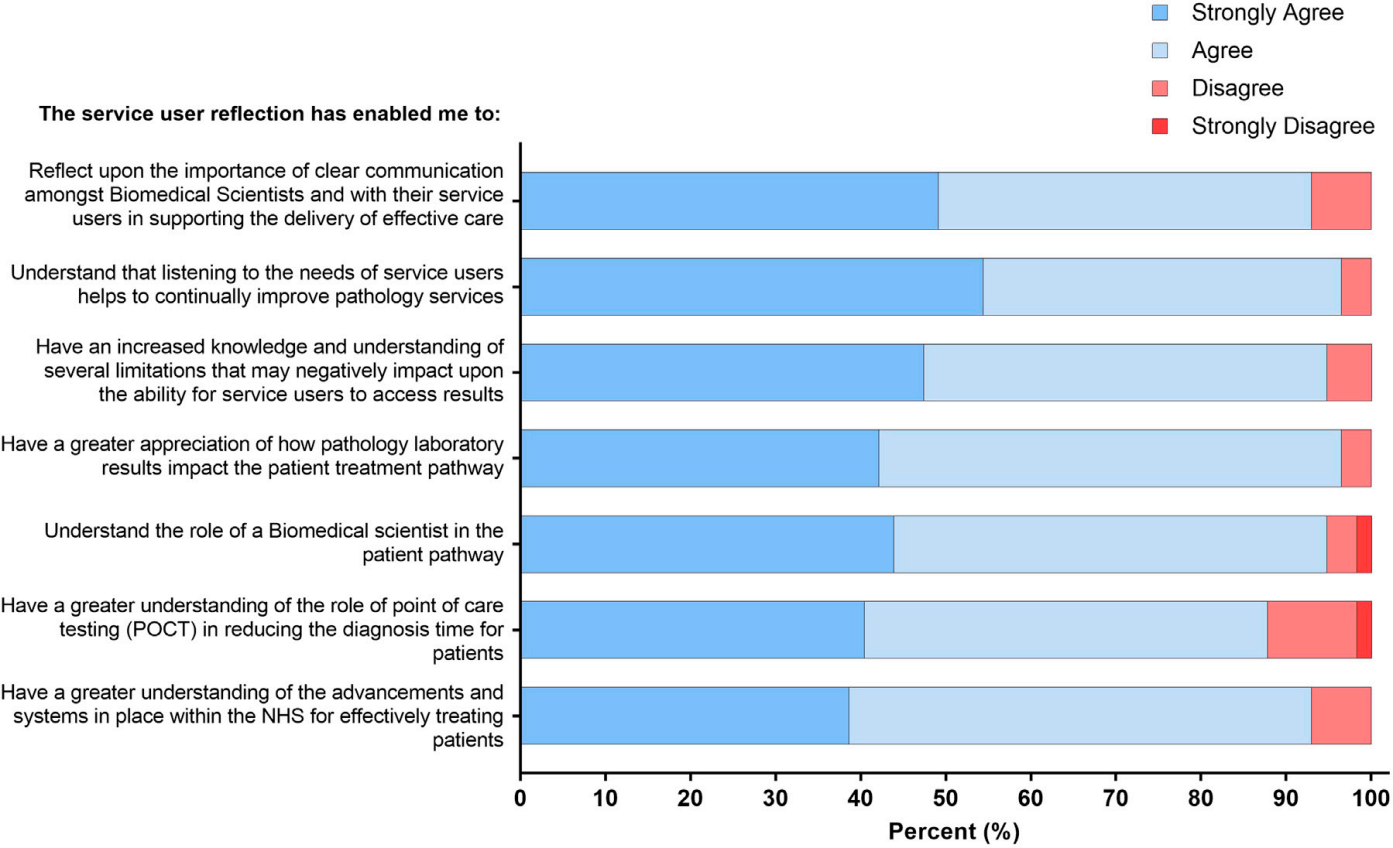
Student responses to statements relating to increased service user awareness and POCT advancements within the NHS pathology laboratories. The response to the four-point Likert scale for each statement is shown as a percentage.

Students wrote a 750-word reflection following the service user event. Post-assessment, over 93% of respondents either “strongly agreed” or “agreed” that the service user reflective assessment reinforced that “*communication amongst Biomedical Scientists*” and “*listening to service users*” are essential in delivering effective patient care through service improvement. Furthermore, 94.8% of respondents either “strongly agreed” or “agreed” that the reflective piece emphasised the importance of both “*the role of the Biomedical Scientist within the pathology laboratory*” within “*the patient treatment pathway*” and the “*limitations that may negatively impact*” the service and ultimately patient results. Lastly, on average, over 90% of respondents either “strongly agreed” or “agreed” that the reflective assessment has increased their understanding of “*POCT and other laboratory advancements within the NHS*” available to reduce diagnostic turnaround times for “*effectively treating patients*” (Figure 3).

### Benefits of Embedding Patients into the Biomedical Science Curriculum

Students were asked for their views regarding the inclusion of patients into the curriculum. Remarkably, 100% of respondents either “strongly agreed” or “agreed” that “embedding patients in the biomedical science curriculum can improve the delivery of healthcare.”

**FIGURE 4:**
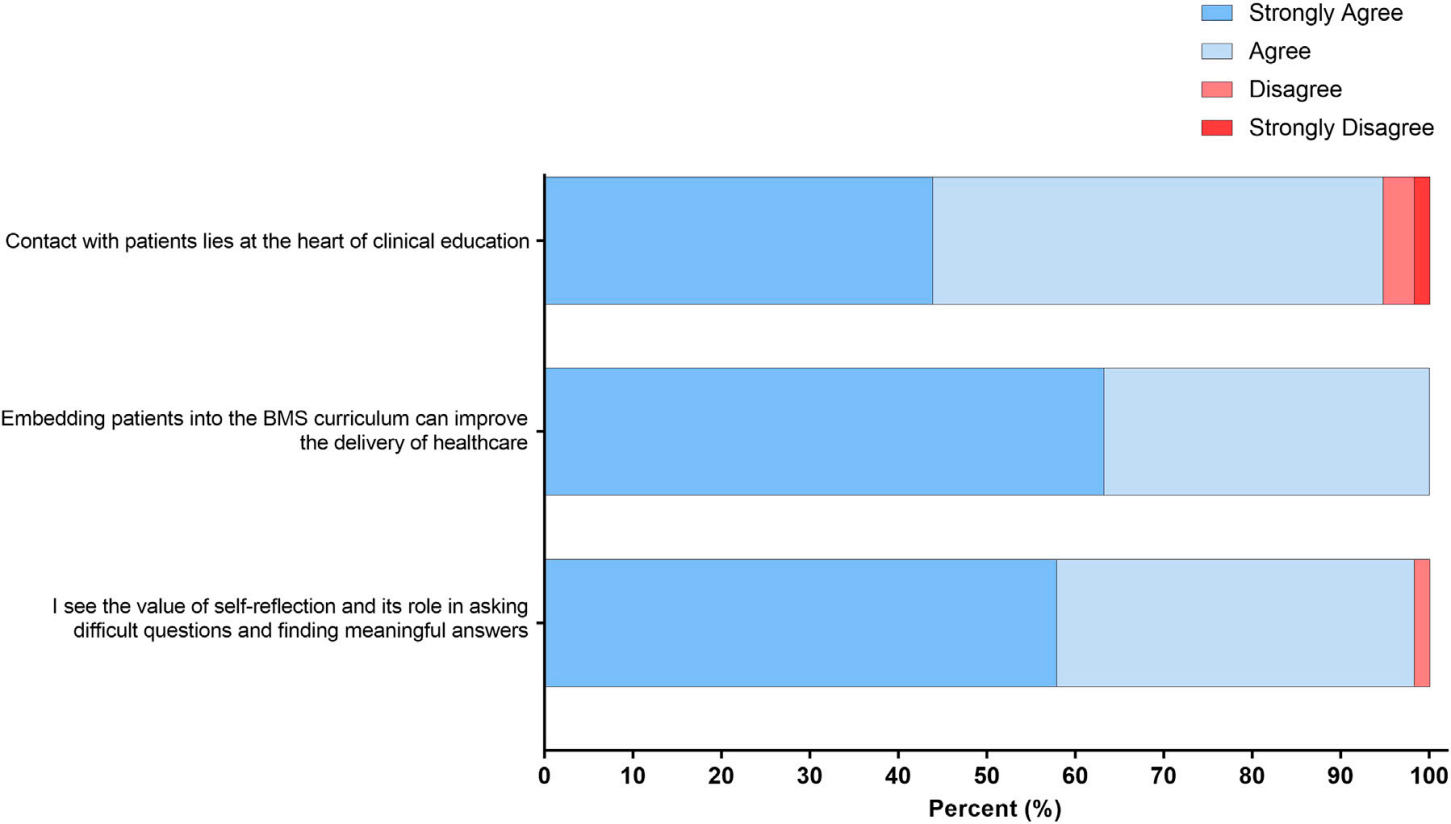
Student responses to statements related to the value of self-reflection and embedding patients into the biomedical science curriculum. The response to the four-point Likert scale for each statement is shown as a percentage.

Furthermore, 94.8% reported that *“contact with the patient lies at the heart of clinical education,”* and 98.3% saw *“the value of self-reflection and its role in asking difficult questions and finding meaningful answers”* (Figure 4).

### Improved Confidence in Reflective Writing for Biomedical Science Students

**FIGURE 5:**
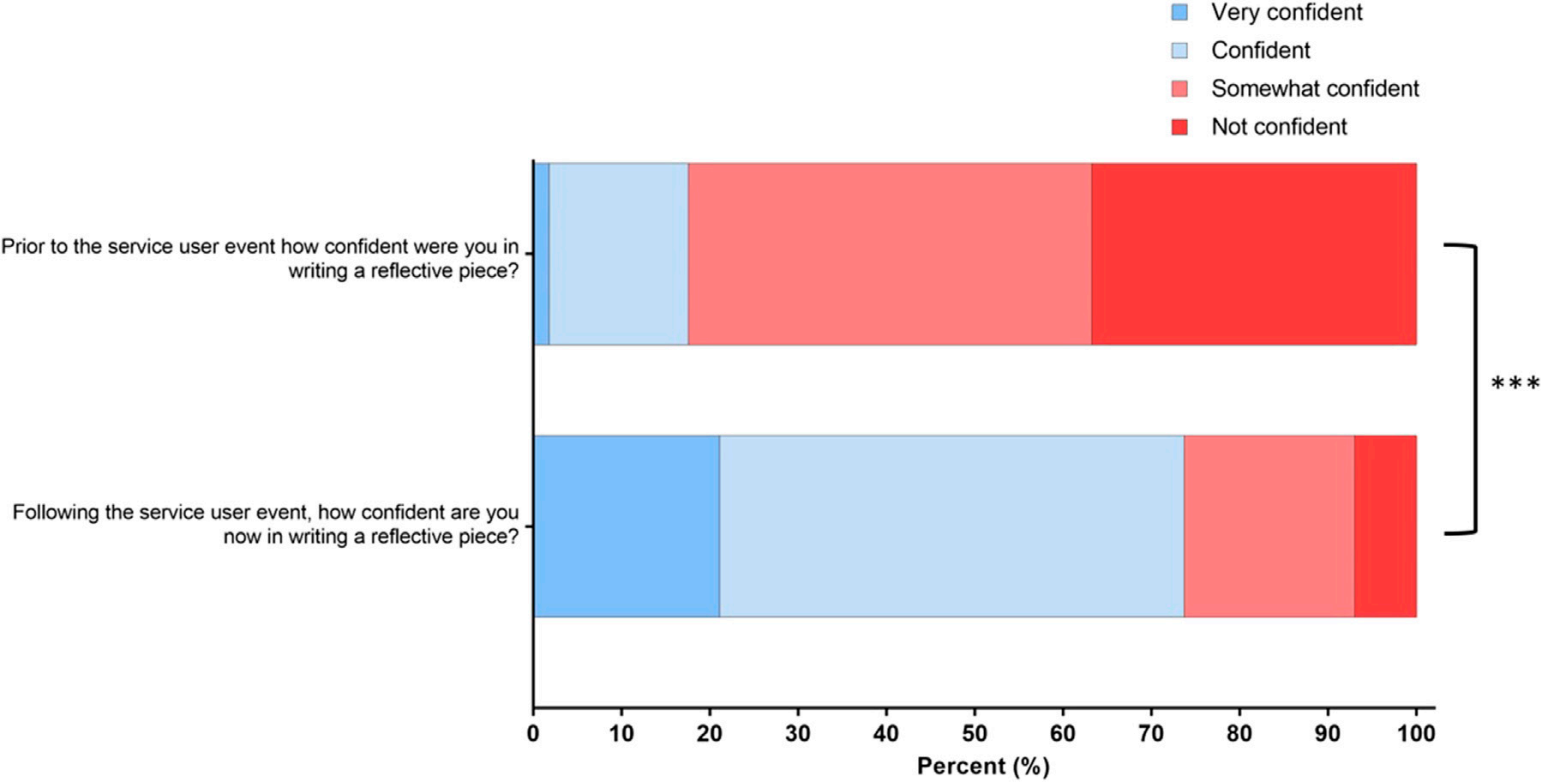
Changes in final-year biomedical science students’ confidence in reflective writing between pre- and post-assessment. Responses to the four-point Likert scale for each statement are shown as percentages ***(*p* < 0.001).

Prior to the completion of the service user reflection, only 17.6% of final-year respondents were either “very confident” or “confident” in reflective writing, compared to 76.3% post-service user assessment (*p <* 0.001) (Figure 5).

### Free Text Responses for Thematic Analysis

**FIGURE 6:**
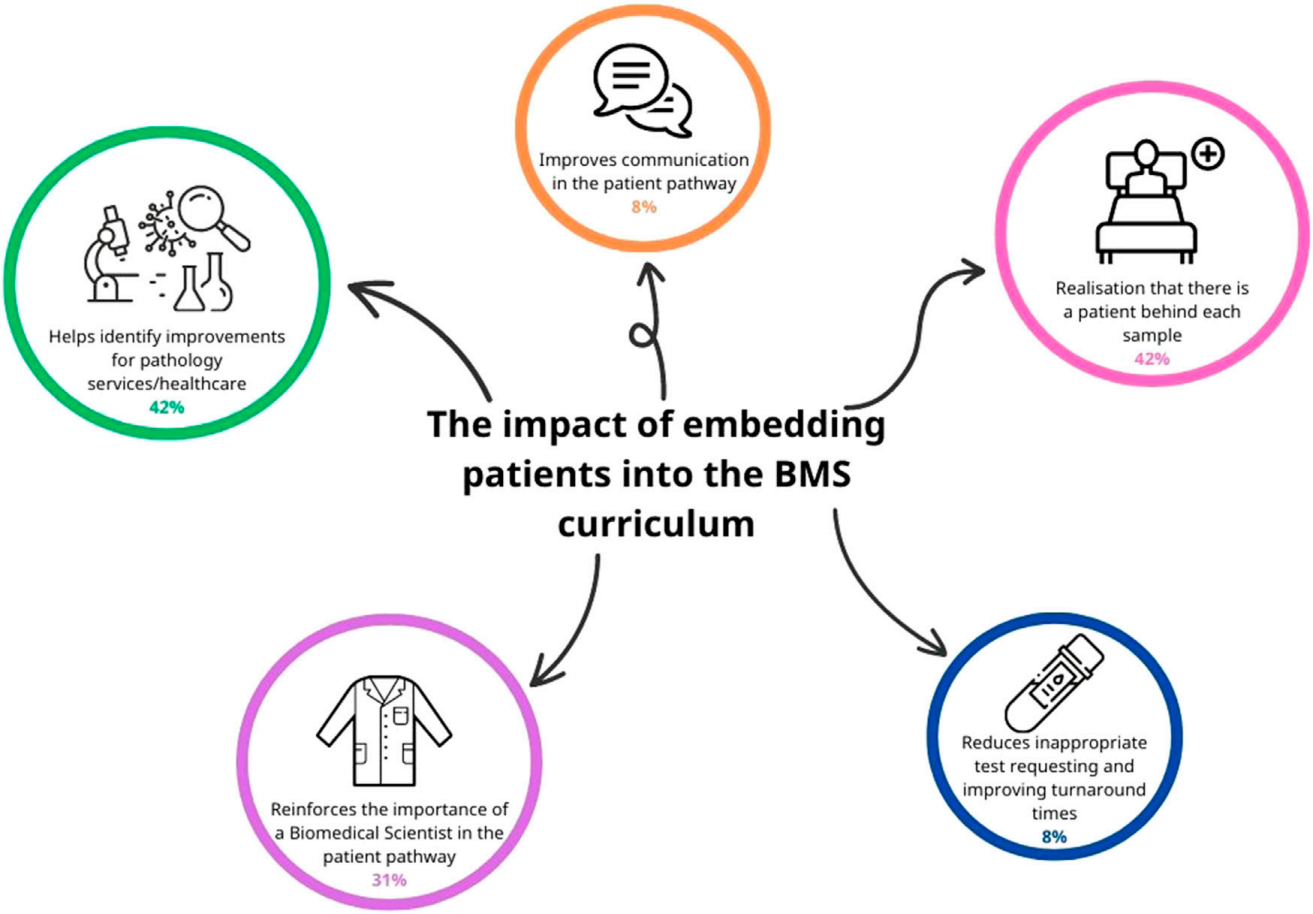
Thematic analysis of open text responses on how embedding service user (patient) involvement in the BMS biomedical science curriculum can improve the delivery of healthcare. Five main themes were identified, and the occurrence of each of these is represented by the size of each circle in the schematic. Some respondents’ open-text responses included more than one theme.

To gauge a better understanding of what students felt about the impact of embedding patients into the biomedical science curriculum, a thematic analysis was conducted. From the responses to the free text question “*Q11. What is the impact of embedding patients into the biomedical science curriculum?”* 46% (*n* = 26) of the respondents answered question 11. Some of the students’ responses fell into multiple themes, and once these were analysed, the five final themes identified were categorised as shown in Figure 6. The most prominent themes identified were 1) realisation that there is a patient behind each sample, 2) helping to identify improvements for pathology services/healthcare and 3) reinforcing the importance of a Biomedical Scientist in the patient pathway.

#### Theme 1: Realisation That There is a Patient Behind Each Sample

A total of 42% of respondents reported that embedding patients in the curriculum reinforced that there is a patient behind each sample and that BMS must always work to a high standard. Listening to the service users reinforced how important each test result is to the patient. Comments included:

*“The event reinforced the importance of pathology results for patients. It can sometimes feel like a process in the laboratory, but each sample is linked to a patient outcome.”* [SIC]

*“Directly including patient experiences in education helps future BMS to recognise the role they play in the patient pathway and emphasises the potential impact they can have on patients, e.g., high standards can save lives, but low standards can cause harm.”* [SIC]

*“One of the core principles of the NHS and public health is ensuring patient is at the "heart" of everything it does. Embedding this principle into student mindset early is integral in ensuring they efficiently perform their particular roles in the future. Future events where we listen to the voice of service users, and their experiences will be progressively beneficial in developing our character so we can best serve our patients and their communities.”* [SIC]

*“Working in laboratories often means little to no patient contact, so hearing patients’ stories helps to reinforce that there’s a patient behind each sample and how what we do in the lab directly impacts patients.”* [SIC]

#### Theme 2: Helps Identify Improvements for Pathology Services/Healthcare

As seen in Theme 1, 42% of respondents also reported that reflecting on service user experiences enabled them to identify improvements for pathology services. These included reducing sample turnaround times, identifying bottlenecks in current service provision, and understanding complex diseases and the impact of complications on patients. Comments included:

*“Patient experiences can help us to identify weaknesses/areas of improvement in the NHS so this has a key role in improving services for its users.”* [SIC]

*“Understanding patient perspectives can help improve services delivered by BMS.”* [SIC]

*“It is important to include patients where necessary into education of healthcare so that we can further our knowledge in terms of a particular disease, its symptoms and any unpredictable complications.”* [SIC]

*“This would improve the time taken to provide patients with relaying test results and any form of diagnostic tests needed to be performed. This would also prevent tests that are not relevant from being tested for that patient as BMS are equipped with much more knowledge within this region.”* [SIC]

#### Theme 3: Reinforces the Importance of the Role of a Biomedical Scientist in the Patient Pathway

The third most common theme, identified by 31% of respondents, was the essential role played by Biomedical Scientists in the diagnosis, monitoring, and treatment of patients. Specific comments made by respondents included:

*“Embedding patients into the curriculum is important for biomedical science students. I feel that it provides perspective of the effects that your decisions can ultimately result in when working as a Biomedical Scientist.”* [SIC]

*“The patient experiences were enlightening. Biomedical Scientists play a crucial role in patient care and should remember how important test results are for individual patients.”*[SIC]

## Discussion

This study aimed to create an opportunity for final-year biomedical science undergraduate students to engage with service users. The “service user event,” accompanied by the reflective writing assessment, involved active student engagement with pathology service users, including both patients and practitioners, to foster a culture of reflective practice among students. The reflective assessment increased students’ awareness of the critical role of pathology laboratory results in ensuring optimal patient care while highlighting strategies to improve existing NHS services to enhance patient experience and outcomes.

### Incorporation of Revised HCPC Standards of Proficiency

The Biomedical Science degree at Aston University is an HCPC-approved course and an IBMS-accredited degree programme. The event and the assessment clearly met and highlighted the importance of the revised 2023 HCPC Standards of Proficiency [3], with an overwhelming number of respondents reporting an increased understanding of how to meet the needs of service users, with key themes being communication, consent, leadership, and use of information (Figure 1). Other studies involving patients within the undergraduate curriculum have reported the importance of creating a diverse learning environment to make education more engaging, powerful, and transformative while ultimately empowering patients [12]. Patients have reported that their involvement in the undergraduate curriculum allows students to hear an alternative perspective in order to better understand their conditions, thus highlighting their empowerment [18].

Our study identified a creative teaching and learning method involving patients and service users [19]. The workshop itself created an environment that was student-directed, participatory, and constructivist by allowing students to openly ask patients about their experiences of [20]. The students produced a 750-word reflective assessment to evaluate patient experiences, which was underpinned by Bloom’s revised framework, which requires students to remember, understand, apply, analyse, and evaluate patient experiences and pathology services [21, 22].

A total of 94.8% of final-year respondents reported an increased understanding of “*public health and prevention of service users’ ill health*” (Figure 1). Students are taught about the clinical presentation, diagnosis, and treatment of haemoglobinopathies as part of the biomedical science curriculum. However, through the service user event, they learned about the experiences of a patient living with beta-thalassaemia major, where the patient highlighted how errors made in the laboratories have substantially impacted their lives. Through a reflective assessment, the students were able to identify instances of good practice and laboratory advancements that could have potentially prevented the transfusion reaction experienced by the patient. Moreover, the students drew attention to emerging technologies such as point-of-care testing (POCT) devices, which offer swift and accurate results, enabling patients to actively manage their conditions and Biomedical Scientists to participate remotely in their care (Figure 2).

Similarly, students are taught the fundamentals of cancer biology as part of their undergraduate degree. However, the inclusion of a patient with a history of a giant cell tumour introduced students to a new malignancy that they would not otherwise have studied. After listening to this patient’s experience, the students acquired new knowledge about the aetiology of a giant cell tumour, the difficulties surrounding the patient’s original misdiagnosis, and the long-term complications that they experienced as a result. After listening to this service user experience, the students were encouraged to undergo the three-stage process of reflection, which includes a recollection of the experience, attending to one’s own feelings, and re-evaluating the experience [14]. Students resonated and empathised with the patient’s difficult experience, and upon re-evaluation, the majority felt that this patient’s experience could have been improved. Suggestions included reducing turnaround times for both pathology and medical imaging tests, improving clinician-patient communication to empower the patient, and increased application of POCT as part of the initial diagnostic testing in an emergency care setting (Figure 3).

The COVID-19 pandemic has hastened changes that were already happening within the biomedical science profession, with regards to greater automation and POCT [23]. The Microbiology Consultant introduced students to the dynamic profession of a Biomedical Scientist, with wider adoption of molecular technologies and laboratories being much more responsive to clinical needs [24]. Furthermore, he showcased how clinical services are also changing and the need for greater efficiency [25]. On reflection, students highlighted that medical staff are often less experienced in understanding and requesting appropriate tests, highlighting the role of the Biomedical Scientist for undergraduate students. Furthermore, students recognised that patient conditions are becoming increasingly complex, requiring more expert advice from laboratories. Students recognised that the traditional role of a Biomedical Scientist will continue to be an important part of the patient pathway in the future while also recognising the need for the role to adapt to reflect technological advances and changing clinical needs (Figure 5). The adaptation of the traditional role of a Biomedical Scientist is already evidenced by the advent of the “Advanced BMS Practitioner” role being introduced into clinical practice [26]. Additionally, the revised HCPC SOPs emphasise the importance of “digital skills and new technologies,” where registrants must be able to “change their practice as needed to take account of new developments, technologies, and changing contexts” [3].

The student survey (Figure 2) identified the importance of effective communication as a key theme. The primary care provider gave examples of the impact of transcription errors in labelling specimens and highlighted the negative impact this had on patients awaiting test results. This situation emphasised to the students how errors that are not communicated to the patient can heighten health-related stress, a theme that was identified by the students as part of their reflective assessment (Figure 5). The patient referral optimisation officer provided insight into the NHS specialist allocation scheme [27] and highlighted to the students some of the challenges that patients can face when enrolled in this scheme. Students reflected on how a negative interaction with a service provider can have a devastating long-term impact on patients. Students saw the value of communicating effectively while asking difficult questions and finding meaningful answers through the use of reflection (Figure 3). As the students identified the importance of effective communication with service users and stakeholders, this workshop met both the HCPC SOPs and the new QAA benchmark statement regarding communication [3, 28]. Other work has highlighted the importance of developing communication and interpersonal skills in undergraduate students [29].

Reflection constitutes a crucial element of continuing professional development (CPD) for healthcare professionals. It is firmly ingrained in the Standards of Proficiency for Biomedical Scientists, serving to safeguard ongoing standards of practice [29]. Following the completion of the reflective assignment, there was a significant increase in students’ confidence when writing reflectively (Figure 4). The ability to reflect is an important and necessary lifelong skill that is highly sought after by employers in an ever-increasingly competitive graduate market. Despite biomedical science programmes effectively educating individuals in highly specialised areas, the transferable skills required, such as critical thinking, effective communication, and the ability to reflect, are often lacking [30]. Biomedical science programmes need to prioritise the inclusion of skill development opportunities through their portfolio of assessments, not only for current students but for them to become lifelong practitioners [30].

**FIGURE 7:**
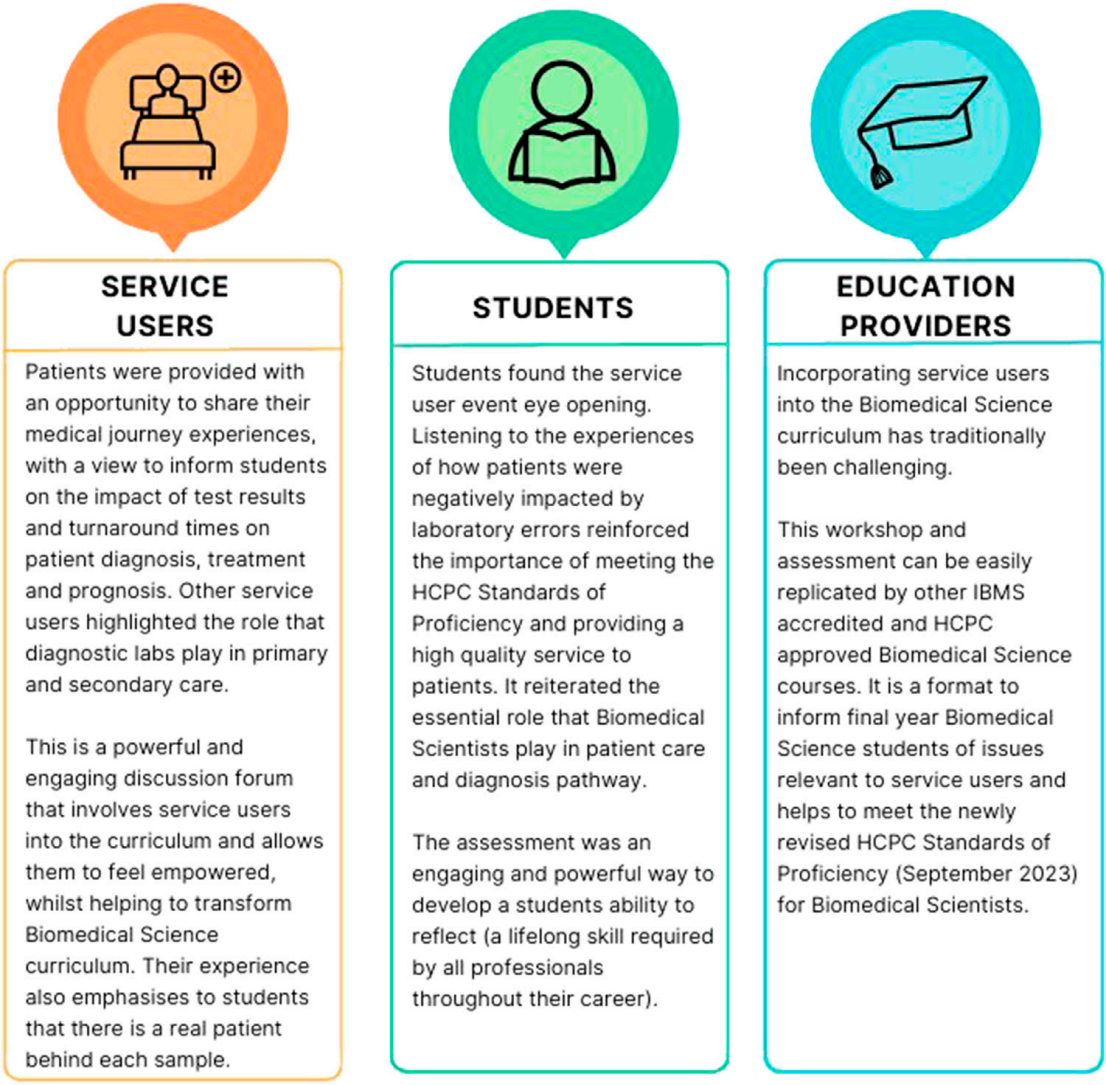
The main benefits and outcomes of the innovative workshop for service users, students, and education providers.

The benefits of patient involvement in the biomedical science curriculum are multifactorial, positively impacting patients, students, and education providers. These benefits are summarised in Figure 7. We hope the workshop can be widely adopted by other higher education institutes.

### Future Work and Study Limitations

Several improvements were suggested by respondents through the open-ended component of the survey. These included increasing the number and diversity of speakers within the workshop to provide a greater overview of the service users of the pathology laboratories within the NHS. In line with this, respondents were in favour of incorporating more patient-focused speakers, as sharing their direct experiences will improve the services within public health. Students also expressed a preference for the opportunity to discuss high-profile cases involving pathology services that affected patient outcomes, such as the case of Dr. Bawa-Garba [31]. Students recognised inequalities in the way that Dr. Bawa-Garba was treated in relation to other medical professionals involved with the patients, highlighting the need for embedding equality, diversity, and inclusion training in education and as part of CPD [32]. The inclusion of ethnically diverse patients and speakers from a wider range of healthcare roles will help to better prepare students for future employment where they will have to work with a more varied population, which will require personalised approaches to their healthcare [33]. In line with other literature, respondents in the current study recognised the value of reflection and expressed an interest in its incorporation as both a formative and summative assessment throughout their biomedical science degree. Previous literature has emphasised the use of curriculum mapping to identify gaps in the curriculum and allow for the constructive alignment of graduate outcomes and assessments [34]. Through the use of curriculum mapping, reflective assignments will be further embedded within the biomedical science curriculum.

In terms of study limitations, at this year’s service user event, we were unable to include Biomedical Scientists and Advanced Practitioners due to timetabling constraints and their availability on the day. In previous events, we have had both professions attend and in the future we will endeavour to include representation from Biomedical Scientists and Advanced Practitioners. Additionally, to enhance attendance and accessibility, we will explore the option of adopting a hybrid approach. This may involve facilitating online participation for professionals, allowing them to connect remotely and interact with students during the event. Furthermore, the overall response rate to the post-workshop survey was 57.5%. While this is higher than the average response rate for similar surveys that usually generate a 30%–40% uptake [35], this could be improved. One suggestion for future work is to collect “before” evaluation data, as this would provide a useful comparison with students learning and skill development following the service user event. In addition, offering financial incentives, such as gift vouchers, would increase the number of survey responses collected, an initiative that is widely used [36]. Finally, the service user event was held face-to-face on campus. Due to the increasing size of the biomedical science student cohorts each year, this can often present logistical challenges, such as finding suitable learning environments and space [17]. Moreover, it can be difficult for patients and service users to travel to university campuses, which may not be local or easily accessible due to their conditions. One potential solution to overcome this is to host an online service user event, although this may come with its own challenges, such as negatively impacting student-service user discussions.

## Conclusion

While professional bodies require programmes to include service users within the biomedical science curriculum, pathology service users are often hard to identify. This large-scale workshop was successful in creating a platform to encompass a range of pathology service users while evoking meaningful discussions between students and these service users. The reflective assessment deepened students’ understanding of the need for efficient NHS pathology services and the crucial role of a Biomedical Scientist in the diagnosis and monitoring of disease. The workshop was an important activity not only in terms of addressing the HCPC SETs in relation to service user involvement but also provided an opportunity to ensure that undergraduate biomedical science students gained an active appreciation of all the current revised HCPC SOPs. Through the reflective assessment, an overwhelming number of students saw the benefits of including pathology service users in the curriculum and developed important transferable skills that are required in graduate careers. We recommend that other IBMS-accredited and HCPC-approved Biomedical Science programmes adopt and embed this innovative workshop into their programmes to help them meet these service users’ standards while fostering important transferable skills in their students.

## Summary Table

### What is Known About This Subject

• Medical and Nursing programmes have successfully included patients in their undergraduate curriculum.
• The revised HCPC SETs require Biomedical Science courses to include service users in the curriculum.
• Including patients into a medically focused curriculum facilitates the development of essential transferable skills.

### What This Paper Adds

• A novel approach to embedding pathology service users and revised HCPC SOPs into the Biomedical Science curriculum.
• Using a pedagogical framework, the reflective assessment encourages students to become reflective practitioners.
• The reflective assessment enhances students’ knowledge and understanding of the impact of pathology results on patients.

## Concluding Statement

This work represents an advance in biomedical science because the innovative workshop developed reflective students who value pathology service users and improvements in NHS service delivery.

## Data Availability

The raw data supporting the conclusion of this article will be made available by the authors, without undue reservation.
