# Supplementary material for: An Innovative Workshop Embedding Pathology Service Users into the Undergraduate Biomedical Science Curriculum
Source: Br J Biomed Sci. 2023 Aug 8;80:11584. doi: 10.3389/bjbs.2023.11584 (PMC10442479; doi:10.3389/bjbs.2023.11584)
Supplement: Supplementary file 1 [file DataSheet3.docx]

# MARK SCHEME FOR THE SERVICE USER REFLECTION

You will need to attend the live service user event to make notes on the speakers that are present.

Following this you are required to submit a **750-word refection** the service user event, include how listening to the voices of service users and taking action can improve the delivery of Biomedical Science.

# Questions to help shape your reflection:

- If Biomedical Scientists listened and responded to the needs of service users, would the pathology service change?
- What specific point of care (POCT) advancements have been made to improve patient care?
- What are the current ‘bottle necks’ in getting results to patients more quickly? How can these be addressed?
- Do you see laboratories being at the backdoor in the near future? Think about recent innovations due to COVID-19
- Are there examples of good practice that can be adopted nationally/internationally?
- What will enable patients to feel more empowered?
- Notice to attain top marks **CRITICAL EVALUATION** is key
- Maximum length **825 words** (this includes the 10% excess condoned by the university) -. Please note any submissions above this have failed the criteria and will not score highly. The word count **does not include** the in-text citations or the reference list. Please cite the word count at the end of your reflection.

The deadline is Week 19, Wednesday 22^nd^ of February, 9am

| **Mark** | **Criteria** |
| --- | --- |
| **80-100** | **All the criteria** for 70-80 and:   1. Critically evaluates a number of areas that could contribute to better patient care 2. Well structured- there is a clear flow between ideas |
| **70-80** | 1. Evidence of deep reflection (analysis, conclusions, action plans) 2. Reflects on at least three speakers and uses a variety of sources/ articles 3. Clear evidence of insight and extra reading beyond the course material with linkage to the ideas discussed during the service user event 4. Well structured 5. Writing is clear and lacking in grammatical errors 6. Citation/ reference list is correctly formatted in accordance to the Harvard style 7. No significant omissions or errors in understanding. |
| **60-70** | 1. There is evidence of deep reflection and the sources are varied 2. Contains no lapses in detail AND evidence of relevant reading beyond material presented 3. Citation/ reference list is correctly formatted in accordance to the Harvard style and is within word limit. 4. Aspects of the reflection are not well structured/written/referenced |
| **50-60** | 1. Most of the reflection is descriptive but there is some evidence of critical thinking 2. Sources are not varied or the reflection is not well structured/ well written/referenced |
| **40-50** | 1. Reflection is descriptive or only about feelings 2. Reflection is not well structured/written/referenced |
| **35-39** | 1. Shows limited evidence of appropriate knowledge and understanding the subject area around service users and improving patient care. 2. Lacks substance or incomplete answer |
| **0-34** | 1. Serious omissions or contains significant errors in understanding the subject area around service users and improving patient care. |
