# Supplementary material for: An Innovative Workshop Embedding Pathology Service Users into the Undergraduate Biomedical Science Curriculum
Source: Br J Biomed Sci. 2023 Aug 8;80:11584. doi: 10.3389/bjbs.2023.11584 (PMC10442479; doi:10.3389/bjbs.2023.11584)
Supplement: Supplementary file 2 [file DataSheet1.PDF]

Online surveys

Service User Event 2023 (copy)

Showing 0 of 0 responses

Showing **all** responses

Showing **all** questions

1 I agree to anonymised direct quotes from the survey being used in publications resulting from the study.

Yes	0

2 I agree to my anonymised data being used by research teams for future research.

Yes	0

3 I agree to take part in this study.

Yes	0

4 Which age category do you belong to?

18-20 years	0
21-24 years	0
25-30 years	0
31-35 years	0
36-40 years	0
41-45 years	0
46-50 years	0
51-55 years	0
56-60 years	0
61-65 years	0
66+ years	0

5 What is your gender identity?

Male	0
Female	0
Non-binary	0
Other	0

6 Which of the categories below best describes your fee status?

Home student	0
EU student	0
Overseas international student	0

7 Have you worked in the NHS in the last three years?

Yes	0
No	0

7.a If yes, please specify your role.

No responses

8 Have you completed an IBMS trainee registration portfolio in a NHS pathology laboratory?

Yes	**0**
No	**0**

9 Following the service user event and reflection assessment I now have increased knowledge and understanding of:

9.1 Public health and prevention of service users' ill-health

Strongly Agree	**0**
Agree	**0**
Disagree	**0**
Strongly Disagree	**0**

Multi answer: Percentage of respondents who selected each answer option (e.g. 100% would represent that all this question's respondents chose that option)

9.2 The role of equality, diversity, and inclusion, with specific importance placed on ensuring practice is inclusive for all service-users

Strongly Agree	**0**
Agree	**0**
Disagree	**0**
Strongly Disagree	**0**

Multi answer: Percentage of respondents who selected each answer option (e.g. 100% would represent that all this question's respondents chose that option)

9.3 The central role of the service-user, including the importance of valid consent and effective communication in providing good care

Strongly Agree	0
Agree	0
Disagree	0
Strongly Disagree	0

Multi answer: Percentage of respondents who selected each answer option (e.g. 100% would represent that all this question's respondents chose that option)

9.4 The importance of leadership at all levels of practice

Strongly Agree	0
Agree	0
Disagree	0
Strongly Disagree	0

Multi answer: Percentage of respondents who selected each answer option (e.g. 100% would represent that all this question's respondents chose that option)

9.5 The need to be able to use information, communication and digital technologies appropriate to practice

Strongly Agree	0
Agree	0
Disagree	0
Strongly Disagree	0

Multi answer: Percentage of respondents who selected each answer option (e.g. 100% would represent that all this question's respondents chose that option)

10 The service user event and reflection assessment has allowed me to:

10.1 Reflect upon the importance of clear communication amongst Biomedical Scientists and with their service users in supporting the delivery of effective care

Strongly Agree	0
Agree	0
Disagree	0
Strongly Disagree	0

Multi answer: Percentage of respondents who selected each answer option (e.g. 100% would represent that all this question's respondents chose that option)

10.2 Has enabled me to understand listening to the needs of service users helps to continually improve pathology services

Strongly Agree	0
Agree	0
Disagree	0
Strongly Disagree	0

Multi answer: Percentage of respondents who selected each answer option (e.g. 100% would represent that all this question's respondents chose that option)

10.3 Have an increased knowledge and understanding of several limitations that may negatively impact upon the ability for service users to access results

Strongly Agree	0
Agree	0
Disagree	0
Strongly Disagree	0

Multi answer: Percentage of respondents who selected each answer option (e.g. 100% would represent that all this question's respondents chose that option)

10.4 Have a greater appreciation of how pathology laboratory results impact the patient treatment pathway

Strongly Agree	0
Agree	0
Disagree	0
Strongly Disagree	0

Multi answer: Percentage of respondents who selected each answer option (e.g. 100% would represent that all this question's respondents chose that option)

10.5 Understand the role of a Biomedical scientist in the patient pathway

Strongly Agree	0
Agree	0
Disagree	0
Strongly Disagree	0

Multi answer: Percentage of respondents who selected each answer option (e.g. 100% would represent that all this question's respondents chose that option)

10.6 Have a greater understanding of the role of point of care testing (POCT) in reducing the diagnosis time for patients

Strongly Agree	0
Agree	0
Disagree	0
Strongly Disagree	0

Multi answer: Percentage of respondents who selected each answer option (e.g. 100% would represent that all this question's respondents chose that option)

10.7 Have a greater understanding of the advancements and systems in place within the NHS for effectively treating patients

Strongly Agree	0
Agree	0
Disagree	0
Strongly Disagree	0

Multi answer: Percentage of respondents who selected each answer option (e.g. 100% would represent that all this question's respondents chose that option)

11 Using the Likert scale, indicate if you agree or disagree with these statements from Clinical sources

11.1 Contact with patients lies at the heart of clinical education

Strongly Agree	0
Agree	0
Disagree	0
Strongly Disagree	0

Multi answer: Percentage of respondents who selected each answer option (e.g. 100% would represent that all this question's respondents chose that option)

11.2 Embedding patients into the BMS curriculum can improve the delivery of healthcare

Strongly Agree	0
Agree	0
Disagree	0
Strongly Disagree	0

Multi answer: Percentage of respondents who selected each answer option (e.g. 100% would represent that all this question's respondents chose that option)

11.a Please expand on your response.

No responses

---

12 Reflection is a rigorous and systematic way of thinking that generates new knowledge by helping us create meaning from our experiences. Following the service user assessment, I now see the value of self-reflection and its role in asking difficult questions and finding meaningful answers.

Strongly Agree	0
Agree	0
Disagree	0
Strongly Disagree	0

---

13 Prior to the service user event how confident were you in writing a reflective piece?

Very confident	0
Confident	0
Somewhat confident	0
Not confident	0

14 Following the service user event, how confident are you now in writing a reflective piece?

Very confident	0
Confident	0
Somewhat confident	0
Not confident	0

15 Please provide any additional comments about what the survey user event reflection has taught you.

No responses

16 Do you have any suggestions for future speakers for the service user event? Do you have any suggestions for how the service user reflection event could be improved?

No responses

17 As a thank you for participating in this study, if you would like the opportunity to be entered into a prize draw to win a Love2Shop monetary voucher, please enter your email address below. The winner will be contacted in April 2023.

No responses
