# Supplementary material for: An Innovative Workshop Embedding Pathology Service Users into the Undergraduate Biomedical Science Curriculum
Source: Br J Biomed Sci. 2023 Aug 8;80:11584. doi: 10.3389/bjbs.2023.11584 (PMC10442479; doi:10.3389/bjbs.2023.11584)
Supplement: Supplementary file 3 [file DataSheet2.docx]

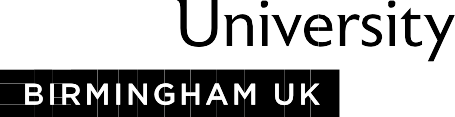


School of Biosciences Assessment Brief

| **Module Code** | BM6PDV |
| --- | --- |
| **Module Title** | Professional Development for Biomedical Scientists |
| **Module Lead** | Amreen Bashir |
| **Assessment Coordinator** | Amreen Bashir |
| **Assessment Title** | Service User Reflection |
| **Assessment Weighting** | 33% of the module |

| **Assessment Brief** | Students need to listen to the talks from the service users. Some of talks will be delivered live (synchronous), whilst most will be pre-recorded clips. Students need to reflect on the talks and include how listening to the voices of service users and taking action to improve problematic areas in healthcare can improve the delivery of Biomedical Science  and improve patient care. |
| --- | --- |
| **Word or Time Limit** | 750 words (+/-10%) |
| **Assessment Criteria** | Students need to show evidence of deep reflection (analysis, conclusions, action plans)  Inclusion of at least three speakers and uses a variety of sources/ articles  Clear evidence of insight and extra reading beyond the course material with linkage to the ideas discussed during the service user event Writing is well structured, clear and lacking in grammatical errors  Citation/ reference list is correctly formatted in accordance to the Harvard style  No significant omissions or errors in understanding. |

*Late Submission will be penalised according to the University Regulations.

*Please pay close attention to the University guidance on Plagiarism and Collusion as well as the Fit to Sit policy.* [https://www2.aston.ac.uk/clipp/documents/Quality/Regulations/202021/used/final-au-rsc-19-](https://www2.aston.ac.uk/clipp/documents/Quality/Regulations/202021/used/final-au-rsc-19-2816-a-regulations-on-student-discipline-202021.pdf) [2816-a-regulations-on-student-discipline-202021.pdf](https://www2.aston.ac.uk/clipp/documents/Quality/Regulations/202021/used/final-au-rsc-19-2816-a-regulations-on-student-discipline-202021.pdf) [https://www2.aston.ac.uk/clipp/documents/Quality/Regulations/au-rsc-18-1855-a-](https://www2.aston.ac.uk/clipp/documents/Quality/Regulations/au-rsc-18-1855-a-general-regulations-for-undergraduate-programmes-2019-20.pdf) [generalregulations-for-undergraduate-programmes-2019-20.pdf](https://www2.aston.ac.uk/clipp/documents/Quality/Regulations/au-rsc-18-1855-a-general-regulations-for-undergraduate-programmes-2019-20.pdf)
